# Supplementary material for: Charge transport in nanoscale vertical organic semiconductor pillar devices
Source: Sci Rep. 2017 Jan 24;7:41171. doi: 10.1038/srep41171 (PMC5259728; doi:10.1038/srep41171)
Supplement: Supplementary Information [file srep41171-s1.pdf]

Supplementary information: Charge transport in nanoscale vertical organic semiconductor pillar devices

Janine G.E. Wilbers[1], Bojian Xu[1], Peter A. Bobbert[1,2], Michel P. de Jong[1], Wilfred G. van der Wiel[1]*.

[1]NanoElectronics Group, MESA+ Institute for Nanotechnology, University of Twente, P.O. Box 217, 7500 AE Enschede, The Netherlands

[2]Molecular Materials and Nano Systems, Department of Applied Physics, Eindhoven University of Technology, P.O. Box 513, 5600 MB Eindhoven, The Netherlands

*W.G.vanderWiel@utwente.nl.

SI-Table 1. Overview of working Au-P3HT-Au devices for different P3HT thicknesses and pillar diameters (s: shorted device; n.c.: non-conductive device; us: unstable device).

P3HT thickness [nm]	Pillar diameter [µm]	Total number of junctions	working junctions	non-working junctions
**5**	2	9	1	7 (s), 1 (us)
	1	11	6	3 (s), 1 (n.c.) 1 (us)
	0.5	16	8	6 (s), 2 (n.c.)
	0.4	1	1	
	0.3	15	4	10 (s), 1 (n.c.)
	0.2	1	1	
**10**	2	29	19	8 (s), 2 (n.c.)
	1	35	22	10 (s), 3 (n.c.)
	0.5	39	26	1, (s), 12 (n.c.)
	0.2	12		10 (n.c.), 2 unstable
**40**	2	8	7	1 (s)
	1	10	9	1 (n.c.)
	0.5	9	7	1 (s), 1 (n.c.)
	0.2	9	6	3 (n.c.)
**100**	2	4	4	
	1	9	7	2 (n.c.)
**total**		**217**	**128**	**89**

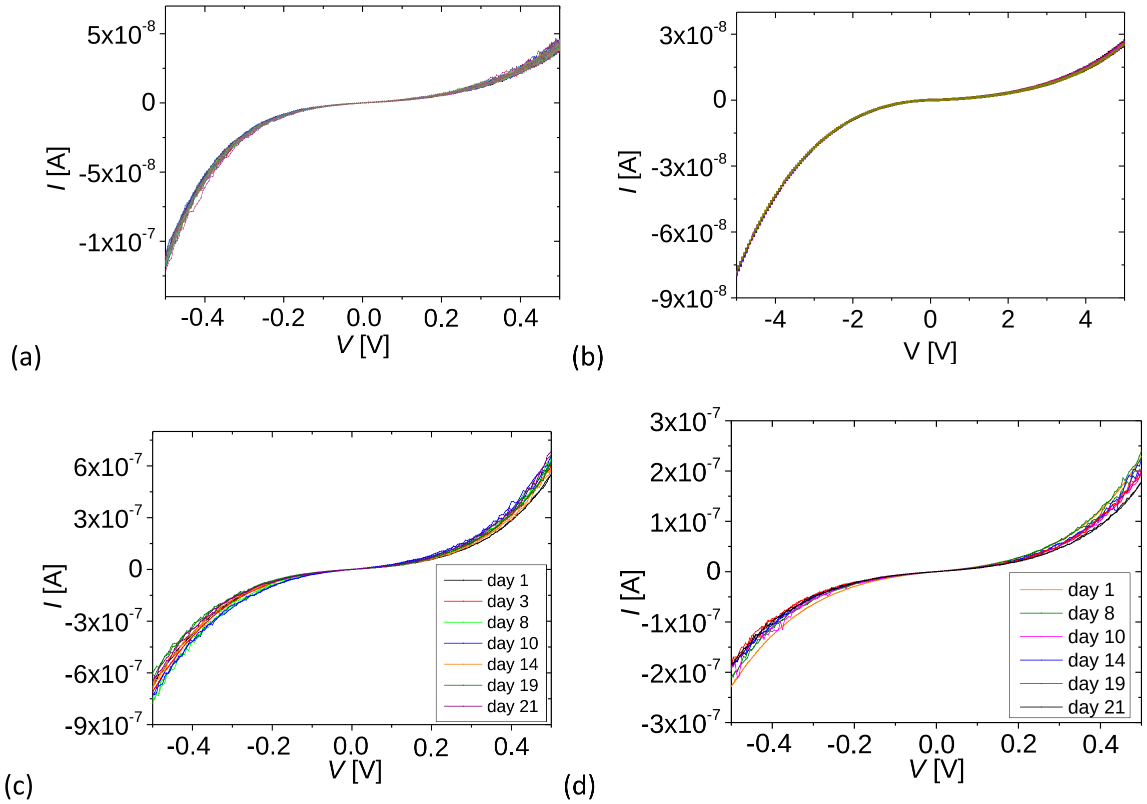

SI-Figure 1. Stability of the Au-P3HT-Au devices measured at room temperature in vacuum (< 10^{-4} mbar) between ± 0.5 V with steps of 0.005 V (a, c, d) and ± 5 V with steps of 0.05 V (b). (a) 40 consecutive I-V sweeps of a device with 10 nm P3HT and a junction diameter of 1 µm; (b) 10 consecutive I-V sweeps of a device with 100 nm P3HT and a junction diameter of 2 µm. I-V sweeps over a time window of three weeks for a device with (c) 5 nm P3HT and a junction diameter of 500 nm and (d) 5 nm P3HT and a junction diameter of 1 µm, respectively.

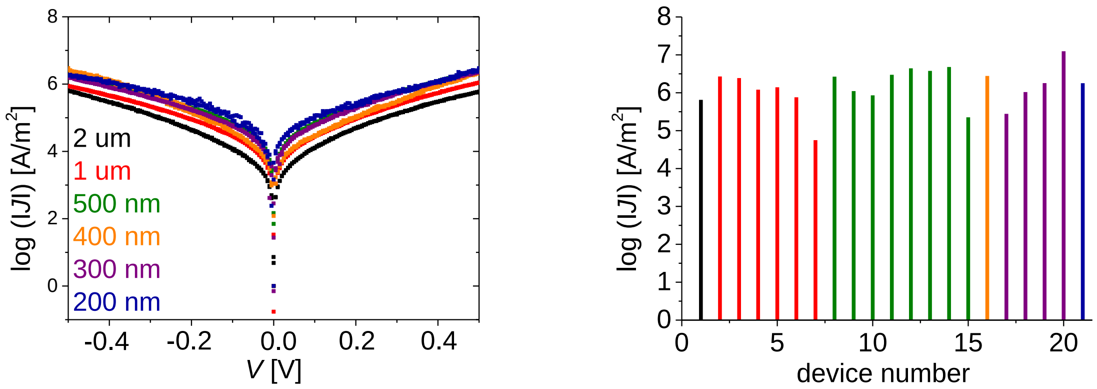
log (|J|) [A/m^2]
V [V]
2 um
1 um
500 nm
400 nm
300 nm
200 nm
device number

(a)

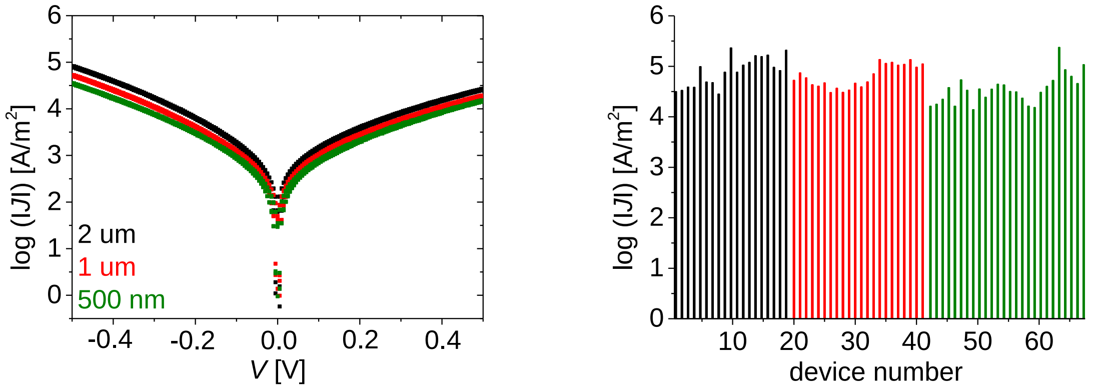
log (|J|) [A/m^2]
V [V]
2 um
1 um
500 nm
device number

(b)

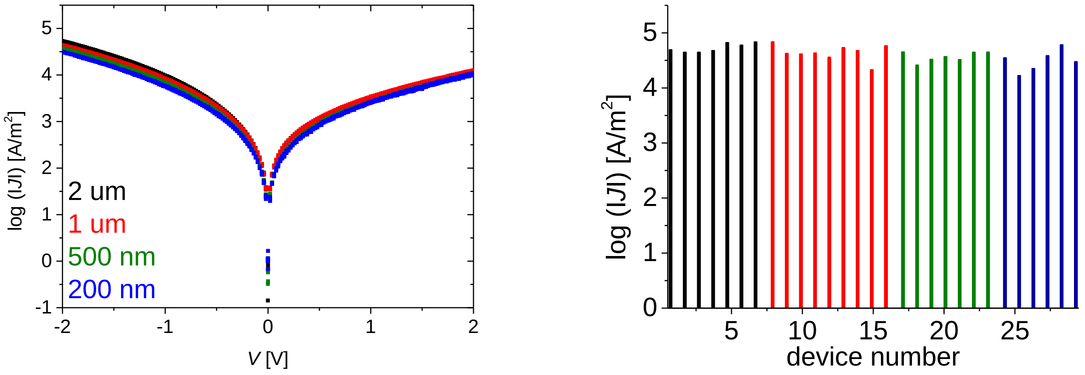
log (|J|) [A/m^2]
V [V]
2 um
1 um
500 nm
200 nm
device number

(c)

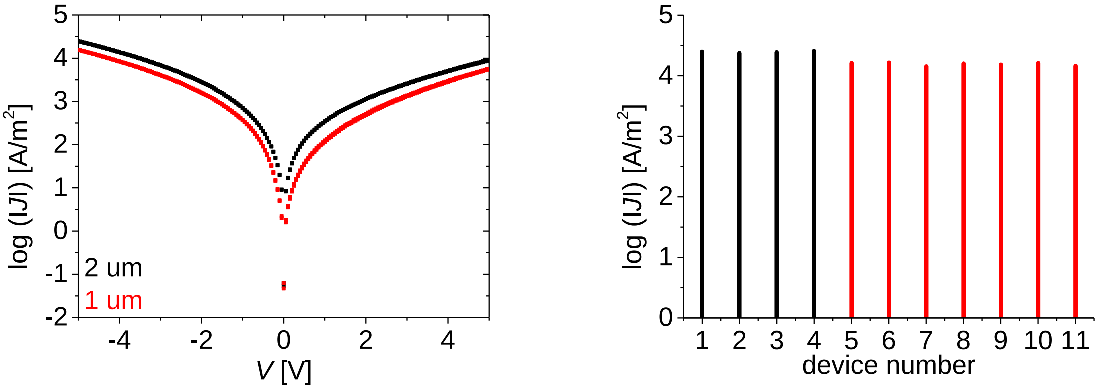
log (|J|) [A/m^2]
V [V]
2 um
1 um
device number

(d)

SI-Figure 2. Electrical characterization of Au-P3HT-Au devices in 2-point configuration at room temperature in vacuum (< 10^{-4} mbar) between ± 0.5 V with steps of 0.005 V for (a) 5 nm P3HT and (b) 10 nm, (c) 40 nm P3HT between ± 2 V with steps of 0.02 V and (d) 100 nm P3HT between ± 5 V with steps of 0.05 V. The curves were obtained by taking the logarithmic average of the current densities measured in devices with the same indicated junction diameter. The graphs on the right side show the corresponding distribution of log (IJI) at -0.5 V.

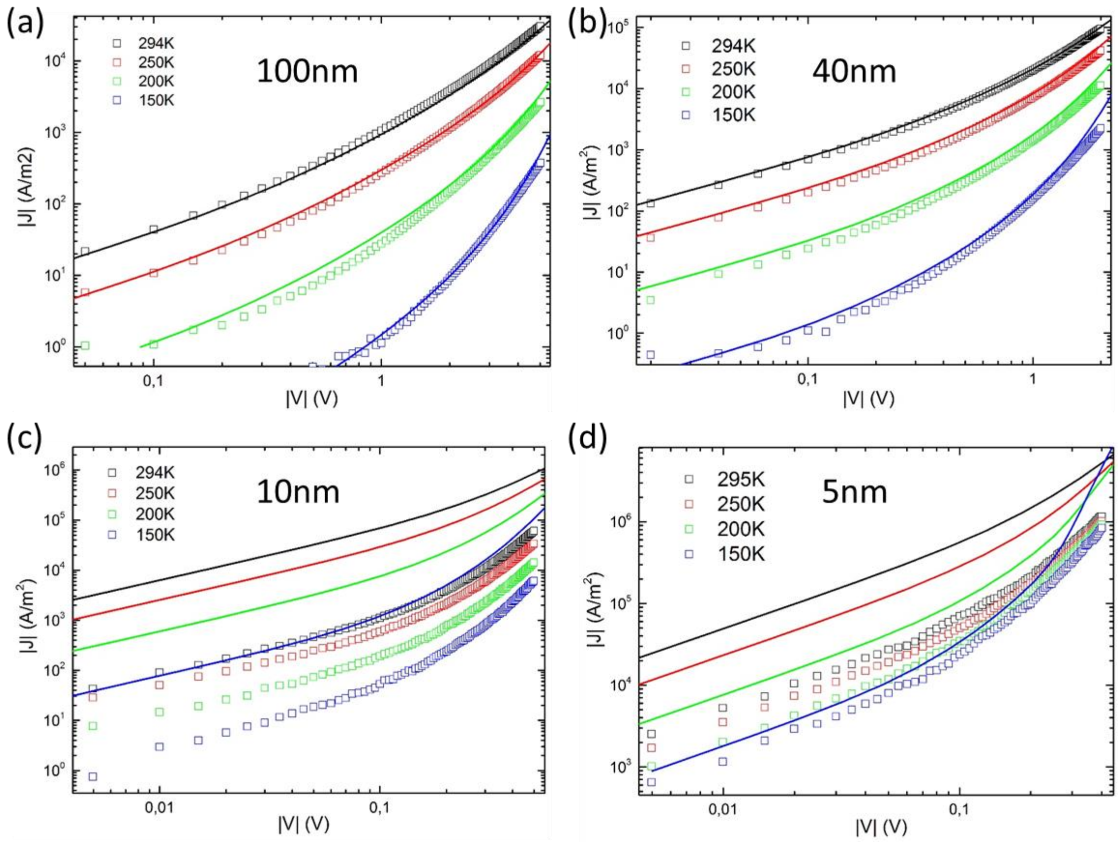

SI-Figure 3. Experimental (symbols) and modelled (lines) current density J versus applied voltage V (negative voltage regime) characteristics of a junction with 2 µm diameter and (a) 100 nm, (b) 40 nm, (c) 10 nm and (d) 5 nm P3HT thickness at various temperatures with an injection barrier of 0.1 eV. The parameters in the drift-diffusion modeling are: room-temperature (294 K) mobility $\mu_0 = 7.3\times10^{-9}$ m^2/Vs, volume density of molecules $N_t = 1.2\times10^{26}$ m^{-3}, width of Gaussian DOS σ = 72.5 meV. The relative dielectric constant used for P3HT is ε_r = 4.4.

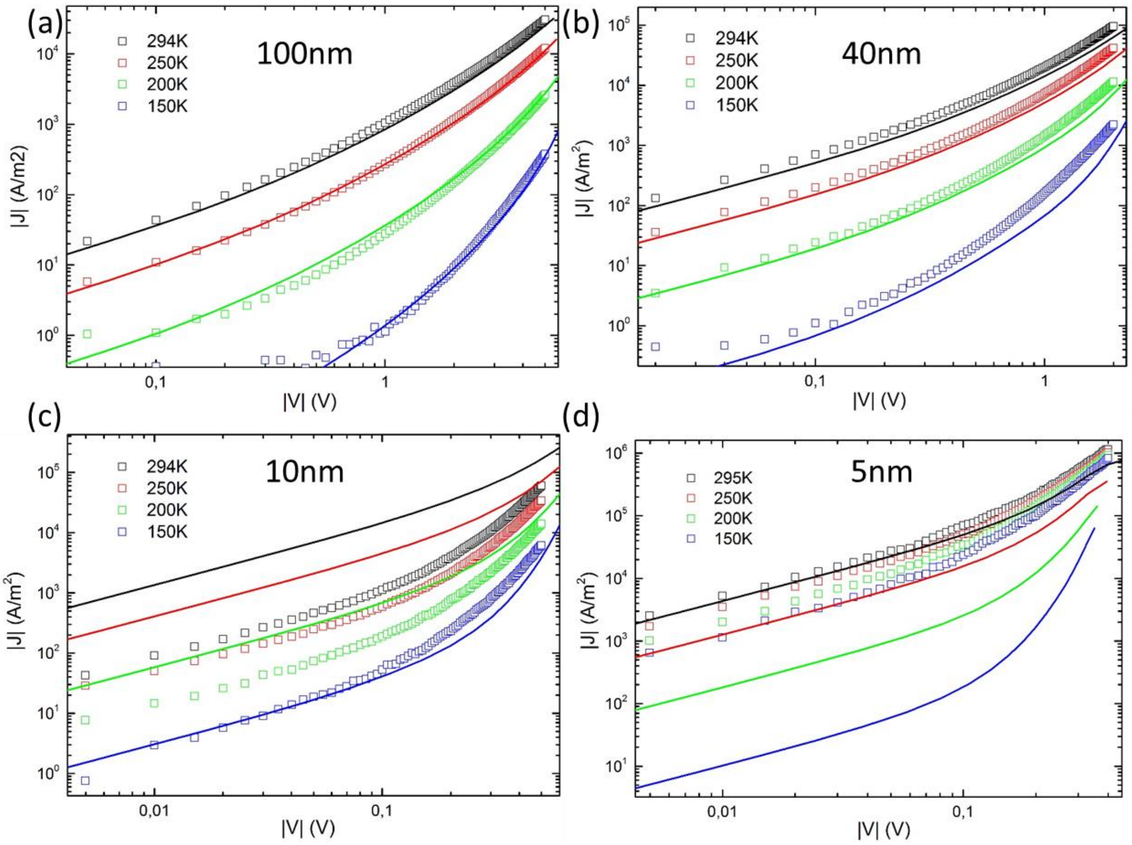

SI-Figure 4. The same as SI-Figure 3, but for an injection barrier of 0.2 eV. The parameters in the drift-diffusion modeling are: $\mu_0 = 8\times10^{-9}$ m^2/Vs, $N_t = 1.0\times10^{26}$ m^{-3}, σ = 71.25 meV.
